# Qualitative analysis and identification of pattern of errors in Clock Drawing Tests of community-dwelling older adults

**DOI:** 10.1590/1980-57642018dn12-020011

**Published:** 2018

**Authors:** Barbara Spenciere, Liana Chaves Mendes-Santos, Christina Borges-Lima, Helenice Charchat-Fichman

**Affiliations:** 1 Pontificia Universidade Catolica do Rio de Janeiro - Psychology - Rio de Janeiro RJ, Brazil.

**Keywords:** Clock Drawing Test, qualitative analysis, older adults, screening, Teste do Desenho do Relógio, análise qualitativa, idosos, rastreio

## Abstract

The Clock Drawing Test (CDT) is a frequently employed screening tool with different scoring systems. Quantitative and semi-quantitative scoring systems, such as Sunderland’s et al. (1989), do not discriminate different error patterns. Thus, the same score can represent a number of different neuropsychological profiles. Therefore, the use of a scoring method that emphasizes qualitative aspects to determine specific error patterns is fundamental.

**Objective:**

To use a qualitative scale to analyze error patterns in the CDTs of older adults who scored 5 in a previous study.

**Methods:**

49 CDTs with score of 5 were analyzed using the qualitative scale. Linear regression and hierarchical and non-hierarchical cluster analyses were performed.

**Results:**

The linear regression showed a significant association between the total score and all the error patterns of the qualitative scale. The hierarchical cluster yielded three groups. However, due to the heterogeneity observed among the groups, a non-hierarchical cluster analysis was performed to better understand the results. Three groups were determined with different neuropsychological profiles and patterns of errors.

**Conclusion:**

The qualitative scoring of the CDT is important when examining and analyzing specific neuropsychological domains in older adults, especially executive functions.

## Resumo

O Teste do Desenho do Relógio (TDR) está entre os instrumentos de rastreio mais utilizados e apresenta diversos métodos de correção e pontuação. Sistemas de pontuação quantitativos e semiquantitativos não discriminam diferentes padrões de erros, como é o caso do método de Sunderland et al. (1989). Dessa feita, uma mesma pontuação pode reunir diferentes perfis neuropsicológicos. Assim, o uso de métodos de correção com ênfase nos aspectos qualitativos, para verificar padrões mais específicos de erro, tornou-se fundamental.

**Objetivo:**

Analisar através de escala qualitativa os padrões de erros no TDR dos idosos cuja pontuação foi 5 em estudo anterior.

**Métodos:**

49 TDR com pontuação 5 foram analisados pela escala qualitativa. Uma regressão linear e análises de cluster hierárquica e não-hierárquicas foram realizadas.

**Resultados:**

A regressão linear mostrou associação significativa entre o resultado total e todos os padrões de erro da escala qualitativa. O cluster hierárquico gerou três grupos. Entretanto devido a heterogeneidade observada entre os grupos, uma análise de cluster não hierárquico foi realizada para melhor entender os resultados. Três grupos foram formados com diferentes perfis neuropsicológicos e padrão de erros.

**Conclusão:**

A pontuação qualitativa do TDR é importante para examinar e analisar domínios neuropsicológicos específicos em idosos, principalmente funções executivas.

The Clock Drawing Test (CDT) has been extolled as a screening tool for dementia. Features such as rapid and easy application are among the reasons for its worldwide clinical use.^1^ There is an extensive discussion about which application and scoring systems produce the most accurate results and no consensus exists on this matter. However, the specificity and the sensitivity of the CDT depend more on the drawing analysis than on its administration.^2^

Methods can be classified into quantitative, semi-quantitative and qualitative.^3^ Authors highlight the semi-quantitative methods proposed by Shulman et al.^4^ and Sunderland et al.,^5^ and the quantitative one proposed by Mendez et al.,^6^ as the most accurate.^2^^,^ 7 Both of these methods are more used to screen dementia. On the other hand, the qualitative scoring methods of the CDT are more used to describe neuropsychological profiles with subtle error patterns.^8^ A qualitative method widely used for this purpose is that of Rouleau et al.^9^

In a previous study,^10^ a specific algorithm method adapted from Sunderland et al.^5^ was employed in a sample of cognitively normal community-dwelling elderly. The initial objectives were to provide a more detailed, specific and quantitative analysis of one of the most used methods of CDT scoring and indicate different aspects of this assessment. Therefore, Mendes-Santos et al.^10^ created the new algorithm with a list of items of more detailed types of errors. It better describes the types of errors of Sunderland’s original hierarchical scale.

The new algorithm of Mende-Santos et al.^10^ found the score of 5 (numbers counter-clockwise or concentrated) to be the most frequent (53.5%), and the mean score of participants was 5.22 (±2.02). By contrast, international and national literature usually describes higher mean scores for cognitively normal elderly. Studies using Sunderland’s scoring method have found scores of 7.5 (±1.9).^5^^,^ 11 ^-^ 16 The score of 5 is below the cut-off point for dementia on the CDT,^13^^,^ 15 including in the original study by Sunderland et al.^5^ which uses a score of 6, where the high frequency of older adults that attained this level was explained by the strict correction done. The method of Sunderland et al.^5^ in its original version had a subjective approach and considered participants with difficulty planning as only those who drew clocks with numbers highly concentrated, unlike in the method of Mendes-Santos et al.^10^ In this other study, participants with both mild and severe deficit in planning were included for a score of 5. The difference illustrated that solely the semi-quantitative scoring method used in this case was insufficient to differentiate whether the difficulty was due to signs of impairments in constructional abilities or executive functions and was unable to grade the difficulty level or pattern of errors.

Given these difficulties with scoring methods of the CDT, a historical review was performed.^17^ Through the history path, improvements in scoring methods using a neuropsychological approach became necessary. Solely the knowledge on the way older adults draw the clock based on final score, without understanding the executive functions involved in the task and the specific types of errors, was no longer sufficient to differentiate groups.^17^

Researchers have demonstrated the advantages of the qualitative approach. Some of these benefits are: differentiation of diagnostic groups^8^^,^ 18 ^,^ 19 such as Mild Cognitive Impairment (MCI), behavioral variant frontotemporal dementia among other conditions;^10^ establishing early differential diagnosis of dementia types,^20^ and also locating lesion sites and differentiating subcortical from cortical cases of stroke.^21^

Despite all the qualitative analysis advantages and growing interest in its administration,^18^ the most used CDT scoring systems in screening dementia are still quantitative and semi-quantitative.^8^^,^ 17 The CDT has not been frequently used to identify older adults with MCI, although the qualitative analysis and the descriptions of subtle errors can contribute in this task.^8^^,^ 18 ^,^ 19

Finally, there is heterogeneity among neuropsychological profiles with cognitive decline and important aspects for diagnoses can be determined by qualitative analysis.^22^ In this context, the use of a qualitative scoring method, taking into account specific error patterns, may be useful to specify the type and level of cognitive decline in different sub-groups.^23^

Therefore, the purpose of this study was to analyze error patterns in the CDTs of older adults without dementia that had a score of 5 in a specific algorithm method adapted from Sunderland et al.^5^^,^ 10 and to verify possible different neuropsychological profiles. The instrument used to examine the drawings was the translated version of the Modified Qualitative Error Analysis of Rouleau^19^ proposed by Fabricio et al.^8^ This method was chosen due to the wide use of the scale and the availability of its translated and adapted version in Brazilian Portuguese.

## Methods

### Participants

The sample comprised forty-nine older adults who attended a social program in Rio de Janeiro. All subjects participated in a previous study^10^ and had a score of 5 (numbers counter-clockwise or concentrated) on the CDT, as scored by a specific algorithm method adapted from Sunderland et al.^5^ The inclusion criteria were being literate (able to read and write), aged 60 or above, absence of dementia (Mini-Mental State Examination - MMSE), having partial dependence and moderate depressive symptoms. The MMSE criteria used was for scores between 0 and 30 that considered the importance of the influence of formal education.^24^^,^ 25 Participants that had uncorrected visual or auditory impairment, endocrine or metabolic abnormalities, impaired performance in hand movements caused by rheumatic diseases or neurological and psychiatric disorders were not included in the sample.

### Materials and procedures

From the protocol of cognitive screening tests previously applied,^10^ the 49 CDTs with scores of 5 were selected randomly. Subsequently, all the CDTs were examined and analyzed using the Modified Qualitative Error Analysis of Rouleau,^19^ translated by Fabricio et al.,^8^ by three different clinical neuropsychologists. The patterns of error analyzed in the scale were Size of the Clock (SC), Graphic Difficulties (GD), Stimulus-Bound Responses (SBR), Conceptual Deficits (CD), Spatial and/or Planning Deficit (SPD) and Perseveration (P). After calculating the total number of errors, the Total Score of the qualitative scale was determined by subtracting the total number of errors from 16.

### Statistical analysis

Descriptive statistical analyses were performed to summarize and elucidate the sociodemographic features of the sample studied. A Kolmogorov-Smirnov test was later used to check for a normal distribution of the variables. A linear regression (stepwise) was performed to assess possible associations between the error patterns of the qualitative scale and its total score. Inter-rater reliability was assessed by comparing CDT scores of the three independent raters.

A hierarchical cluster, employing the Chi-square method as a dissimilarity measure, was used to group the participants with similar error patterns and total score. A non-hierarchical cluster analysis (K-means) was then administered to confirm the results. Chi-square tests were then performed to compare differences between the cluster groups and verify the level of significance between the variables. The level of significance was set at 1%, i.e. p≤0.01.

## Results

The demographic and cognitive characteristics of the sample are given in Table 1. Table 1 also shows that participants with moderate depressive symptoms (score >5 on the GDS) and partial dependence (score <21 on Lawton’s Scale) were included in the study. Regarding depressive symptoms, 3 depressed participants (6.1%) and 17 elderly with partial dependence (34.7%) were part of the sample.

**Table 1:** Demographic and cognitive characteristics of the sample.

	N	Mean (SD)	Minimum score	Maximum score
Female/Male	45/4	–	–	–
Age (years)	49	72.1 (6.3)	60	84
Education (years)	49	9.9 (4.0)	3	18
MMSE	49	24.6 (3.0)	18	30
CDT Sunderland	49	5 (0)	5	5
CDT qualitative*	49	11.4 (1.2)	8	14
GDS	49	1.81(1.98)	0	8
Lawton’s Scale	49	20.42(0.93)	18	21

N: number, SD: standard deviation.

* Fabricio et al. (2014). GDS: Geriatric Depression Scale.

**Figure 1:**
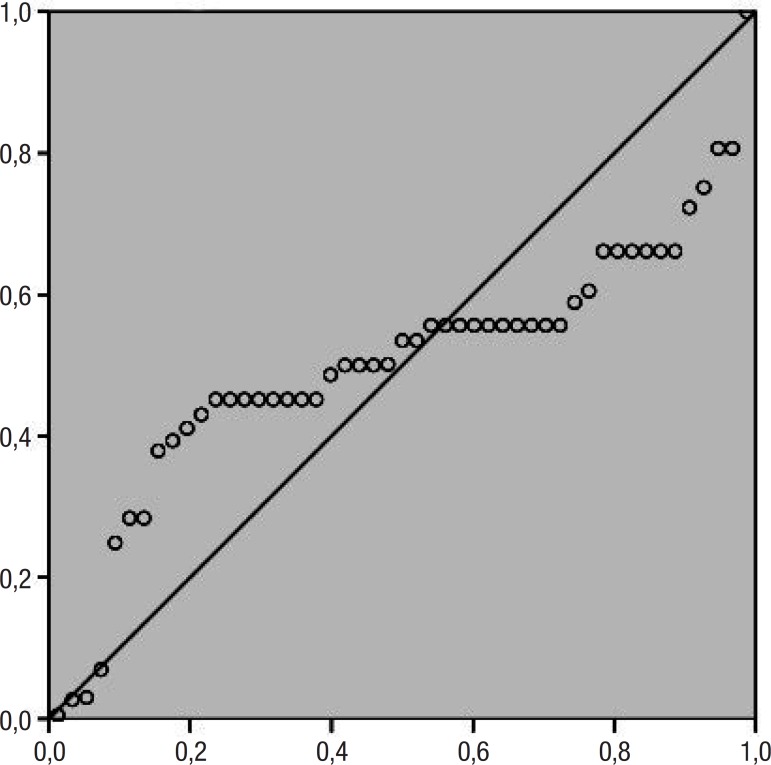
Linear regression.

The linear regression showed a significant association between total score and all error patterns on the qualitative scale, as depicted in Figure 1. Regarding the types of errors, the sample comprised clock drawings characterized by numbers counter-clockwise or concentrated. Therefore, all drawings had spatial and/or planning deficits (100%). However, the frequency of other patterns of errors was also described, as presented in Table 2.

**Table 2:** Frequency of errors in the Modified Qualitative Error Analysis of Rouleau

Types of errors	Frequency	%
**Size of the clock**		
Small	29	40.8
Large	0.0	0.0
**Stimulus-bound response**		
Time in print or digital	1	2.0
Pointers tied to stimulus	2	4.1
**Graphic difficulties**		
Mild	34	69.4
Moderate	11	22.4
Severe	0	0.0
**Conceptual deficits**		
Misrepresentation of the Clock itself	1	2.0
Misrepresentation of the time	46	93.9
Numbers Out of Order or Missing	0	0.0
**Spatial/planning deficits**		
Neglect of the left hemi-space	0	0.0
Deficit in spatial planning of numbers	48	98.0
Deficit in planning	34	69.4
Numbers written outside the clock face	0	0.0
Numbers written counter-clockwise	0	0.0
**Perseveration**		
Perseveration of hands	5	10.2
Perseveration of numbers	3	6.1

The investigation of inter-rater reliability of the CDT scored by the Modified Qualitative Error Analysis of Rouleau^19^ showed mean variation in qualitative total score of between 11.12 and 11.41. Pearson’s correlation analysis was performed between the scores determined by the three independent raters (p<0.01): 1 and 2 (r=0.87), 1 and 3 (r=0.80), 2 and 3 (r=0.82). Raters’ agreement was highly significant (in all cases, p<.001). Weighted Kappa scores verified the level of agreement, which were all significantly above chance (p<0.001).

**Figure 2:**
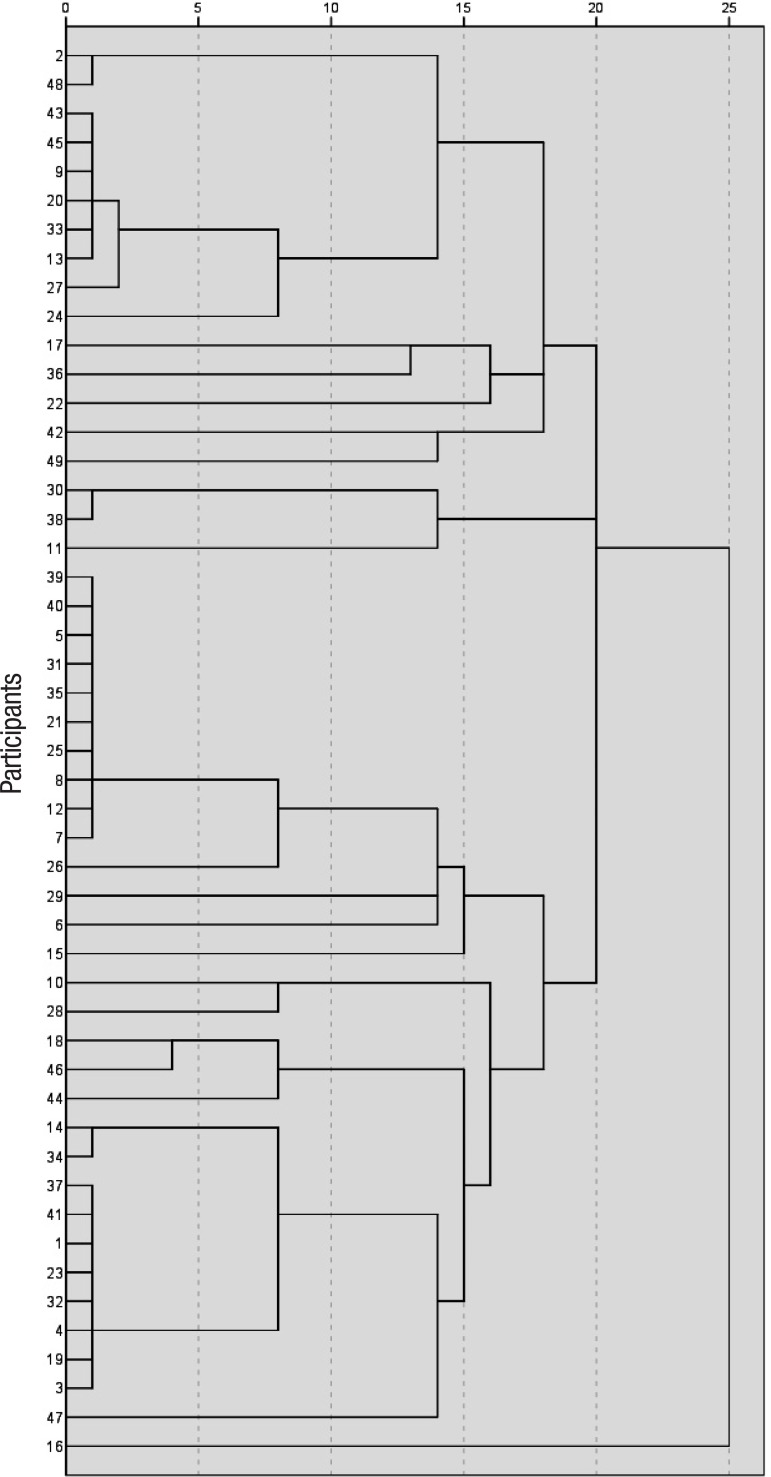
Hierarchical cluster dendrogram.

Figure 2 shows a dendrogram of the hierarchical cluster performed with the Chi-Square Method as a dissimilarity measure. The cluster yielded different groups at first (five), second (ten), third (fifteen), fourth (twenty) and fifth (twenty-five) levels. At level 1, heterogeneity was predominant and groups could not be distinguished. By level 2, similar participants were featured and seven groups verified. Between level 3 and 4, three broader groups could be distinguished. At level 4, all the groups previously distinguished could be pooled. Finally, at level 5, participants with different features joined the other groups. Heterogeneity was evident in this cluster analysis.

Due to the heterogeneity observed through the groups yielded by the hierarchical cluster shown in Figure 2, a non-hierarchical cluster analysis (K-means) was performed to better understand the results. This second cluster analysis distinguished three groups, as described in Table 3. Comparing groups, small size of the clock, graphic difficulties (mild and moderate), planning deficits without a specific pattern and perseveration of hands were the types of errors that differed between them (Table 3).

**Table 3:** Cluster centers of pattern of errors, total score.

	Group 1 (N=37)	Group 2 (N=8)	Group 3 (N=4)
**Size of the clock**			
Small	1	0	1
Large	0	0	0
**Stimulus-bound response**			
Time in print or digital	0	0	0
Pointers tied to stimulus	0	0	0
**Graphic difficulties**			
Mild	1	1	0
Moderate	0	0	2
Severe	0	0	0
**Conceptual deficits**			
Misrepresentation of the Clock itself	0	0	0
Misrepresentation of the time	1	1	1
Numbers Out of Order or Missing	0	0	0
**Spatial/planning deficits**			
Neglect of the left hemi-space	0	0	0
Deficit in spatial planning of numbers	1	1	1
Deficit in planning	1	0	1
Numbers written outside the clock face	0	0	0
Numbers written counter-clockwise	0	0	0
**Perseveration**			
Perseveration of hands	0	0	1
Perseveration of numbers	0	0	0
Total Score	11	13	9

**Figure 3:**
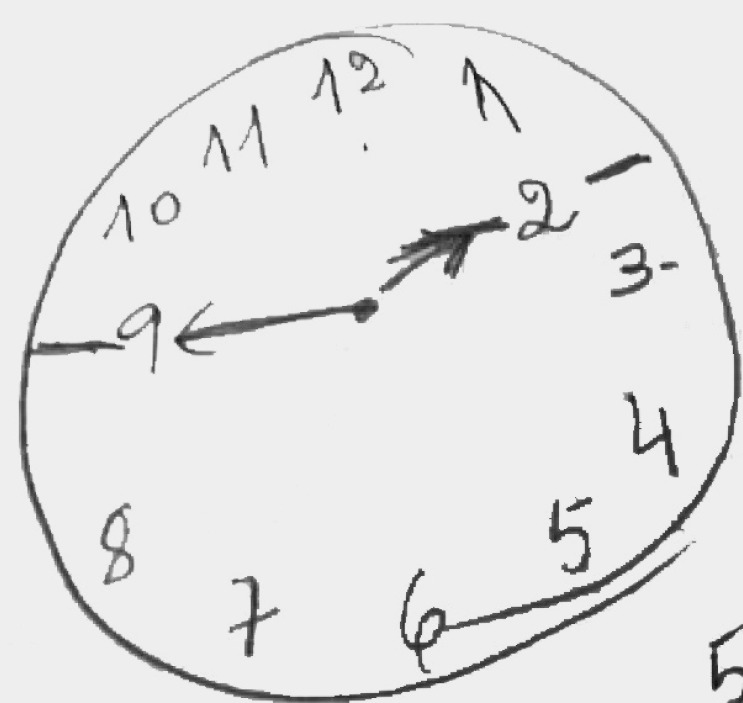
Group 1.

**Figure 4:**
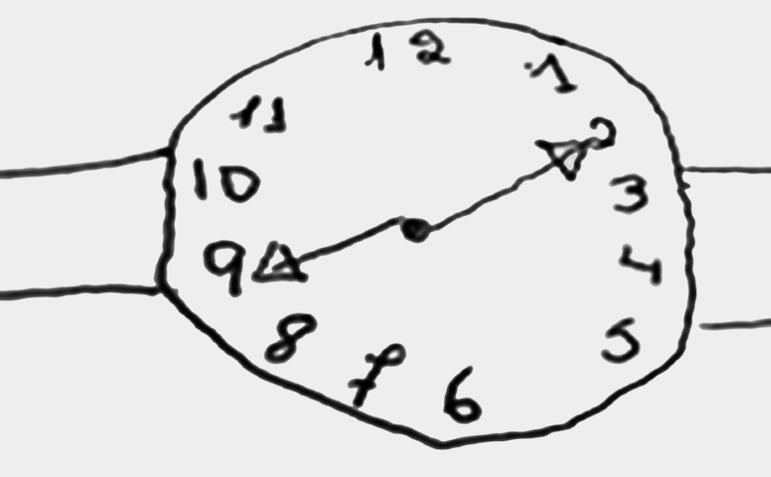
Group 2.

**Figure 5:**
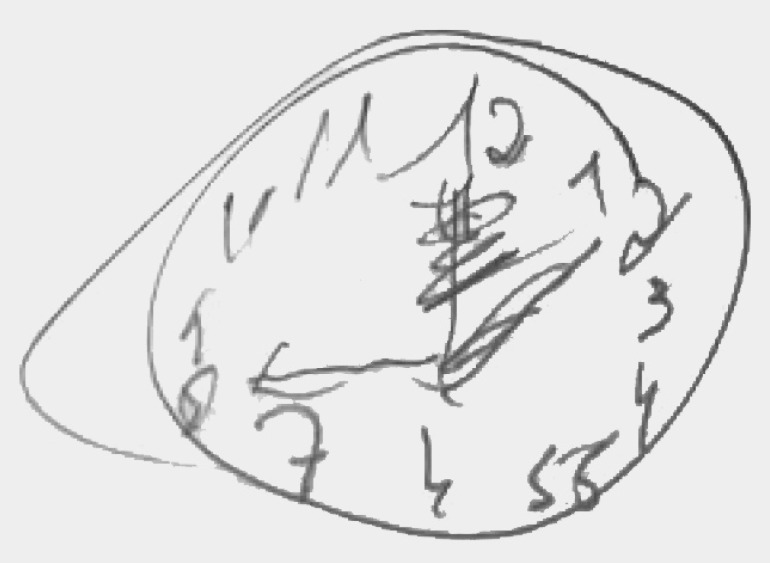
Group 3.

Table 3 shows that different neuropsychological profile groups differed in levels of difficulties and pattern of errors. Among a sample of older adults that had the same score of 5 by the method of Sunderland et al.,^5^ groups with different levels of difficulties were distinguished. Figures 3, 4 and 5 provide CDT examples that illustrate each group.

## Discussion

The aim of this study was to analyze error patterns in CDTs and verify whether different neuropsychological profiles could be distinguished. The sample was composed of cognitively normal older adults that all scored 5 (numbers counter-clockwise or concentrated) in a previous study^10^ and the instrument used to describe the types of errors was the Modified Qualitative Error Analysis of Rouleau,^19^ devised by Fabricio et al.^8^

Fabricio et al.^8^ also found a high prevalence of planning deficits in their sample of healthy older adults, where this was the most frequent type of error in the study. This data matches the results found in the present sample, part of a previous study by Mendes-Santos et al.^10^

The initial objective was to understand why more than half of the participants in the cited study had a score of 5, which is below the cut-off point for dementia, considering that the semi-quantitative method^5^ possibly does not differentiate executive from constructive deficits. The purpose of a qualitative scale was to attempt to differentiate these deficits and identify subgroups of elderly with different levels of difficulty and subgroups with different error patterns.

Considering all subjects in the present sample had planning deficits, the discussion will address the other frequent pattern of errors. In general, conceptual deficits (misrepresentation of time), graphic difficulties (mild) and size of the clock (small), respectively, were the other types of errors with higher frequency. Similar results were found in the study by Fabricio et al.^8^ However, the same study analyzed a sample with cognitive decline that had a different distribution of types of error frequency. Conceptual deficits and planning deficits, followed by graphic difficulties, size of the clock, perseveration and stimulus-bound response were the most frequent types of errors.

A lower performance for clock numbers and hands (conceptual deficit) occurs among healthy older adults.^8^ This type of error increases significantly in mild cognitive impairment (MCI) and becomes even more frequent in patients with AD.^8^^,^ 19 ^,^ 20 However, in this study, there was a high presence of conceptual deficits, as mentioned, especially in time representation. This might have occurred for two reasons. Firstly, because the study sample included elderly with depressive symptoms and partial dependence. Secondly, because of the strict scoring of the algorithm used by the specialists to score the test.^10^

A high prevalence of graphic difficulties and size of Clock pattern of errors were also present in studies performed with healthy samples.^8^^,^ 19 ^,^ 26 Although this study sample comprised non-healthy individuals, none had dementia, which can justify the similar results.

On the other hand, stimulus-bound response and perseveration errors were less frequent in this sample. The literature confirms that both stimulus-bound response and perseveration errors are not commonly committed by older adults without dementia.^8^^,^ 19 ^,^ 20 ^,^ 26 ^,^ 27

Among a sample with a semi-quantitative score of 5,^5^ the hierarchical cluster yielded different neuropsychological profile groups characterized by different types of error. At the first level, greater heterogeneity was observed within the sample. On the other hand, as groups were pooled, similarities could be observed.

Different neuropsychological profiles were evident. There were similarities among the groups (Table 3) represented by the absence of some types of errors and presence of the deficit in planning of numbers and misrepresentation of the time. Besides patterns of error, different levels of deficits could also be distinguished. Group 3 was the smallest but the most impaired group. It featured small clock drawing, moderate graphic difficulties, planning deficits without a specific pattern and perseveration for hands. By contrast, group 1 was the largest group, had less graphic difficulties and no perseveration for hands. Finally group 2 was the least impaired.

Regarding spatial/planning deficits, group 2 had a lower frequency of this type of error. On the other hand, the two other groups could not be differentiated solely using the qualitative scale. It is important to mention that the scale only scored the final drawing. As the best methods for determining planning strategies are those that analyze the whole process of construction of the drawing,^28^ this might explain why specific aspects of planning deficits could not be described.

Comparing healthy older adults to those with cognitive decline, the frequency of all types of error increases.^8^^,^ 19 ^,^ 26 This may explain the different levels of difficulties among the sample and highlights the importance of qualitative analyses of the CDT as a tool for neuropsychological assessment.

As the exclusion criterion of the study was based on the MMSE, this sample likely comprised older adults without dementia. However, considering the type and level of errors of Group 3 and the presence of participants with partial dependence and depressive symptoms, the study might have included individuals with MCI.

These results support the initial hypothesis that the use of a qualitative scoring method that takes into account specific error patterns could be useful for specifying e type and level of cognitive decline in different subgroups. Thus, different neuropsychological profiles can be described in a sample of older adults without dementia.

The neuropsychological approach in scoring systems of the CDT is an important aspect, as the quantitative and semi-quantitative scoring methods alone cannot differentiate groups.^17^ It is evident that the Modified Qualitative Error Analysis of Rouleau^19^ can describe specific patterns of errors and neuropsychological profiles in older adults from the community. Thus, the use of the qualitative scale can be valuable as a complementary tool to the semi-quantitative scale when scoring the CDT.

Although the scale was also useful for discriminating types of errors of executive functions from constructional abilities, the type of analysis that scores the drawing only without considering the process and the sequence used in the construction, is not the best method for determining planning strategies. This information could be a complementary aspect to allow a better qualitative description of executive functioning in elderly. Therefore, further studies with a larger sample or a study that describes the construction strategies of drawing a clock could help understand planning and organization features.

## References

[1] Hubbard EJ, Santini V, Blankevoort CG, Volkers KM, Barrup MS, Byerly L (2008). Clock drawing performance in cognitively normal elderly. Arch Clin Neuropsychol.

[2] Shulman KI (2000). Clock-drawing: is it the ideal cognitive screening test?. Int J Geriatr Psychiatry.

[3] Ehreke L, Luck T, Luppa M, König HH, Villringer A, Riedel-Heller SG (2011). Clock drawing test: screening utility for mild cognitive impairment according to different scoring systems: results of the Leipzig Longitudinal Study of the Aged (LEILA 75+). Int Psychogeriatr.

[4] Shulman KI, Gold DP, Cohen CA, Zucchero CA (1993). Clock-drawing and dementia in the community: a longitudinal study. Int J Geriatr Psychiatry.

[5] Sunderland T, Hill JL, Mellow AM, Lawlor BA, Gundersheimer J, Newhouse PA (1989). Clock drawing in Alzheimer's disease: a novel measure of dementia severity. J Am Geriatr Soc.

[6] Mendez MF, Ala T, Underwood KL (1992). Development of scoring criteria for the clock drawing task in Alzheimer's disease. J Am Geriatr Soc.

[7] Storey JE, Rowland JTJ, Basic D, Conforti DA (2001). A comparison of five clock scoring methods using ROC (receiver operating characteristic) curve analysis. Int J Geriatr Psychiatry.

[8] Fabricio AT, Aprahamian I, Yassuda MS (2014). Qualitative analysis of the Clock Drawing Test by educational level and cognitive profile. Arq Neuropsiquiatr.

[9] Rouleau I, Salmon DP, Butters N, Kennedy KC, McGuire K (1992). Quantitative and qualitative analyses of clock drawings in Alzheimer's and Huntington's disease. Brain Cogn.

[10] Mendes-Santos LC, Mograbi D, Spenciere B, Charchat-Fichman H (2015). Specific algorithm method of scoring the Clock Drawing Test applied in cognitively normal elderly. Dement Neuropshycol.

[11] Cecato JF, Fiorese B, Montiel JM, Bartholomeu D, Martinelli JE (2012). Clock Drawing Test in Elderly Individuals with Different Education Levels Correlation with Clinical Dementia Rating. Am J of Alzheimers Dis Other Demen.

[12] Hamdan AC, Hamdan EMLR (2009). Teste do desenho do relógio: desempenho de idosos com doença de Alzheimer. RBCEH.

[13] Kirby M, Denihan A, Bruce I, Coakley D, Lawlor BA (2001). The clock Drawing Test in primary care: sensitivity in dementia detection and specificity against normal and depressed elderly. Int J Geriatr Psychiatry.

[14] Lee H, Swanwick GRJ, Coen RF, Lawlor BA (1996). Use of the clock drawing task in the diagnosis of mild and very mild Alzheimer's disease. Int Psychogeriatr.

[15] Nunes PV, Diniz BS, Radanovic M, Abreu ID, Borelli DT, Yassuda MS, Forlenza OV (2008). CAMcog as a screening tool for diagnosis of mild cognitive impairment and dementia in a Brazilian clinical sample of moderate to high education. Int J Geriatr Psychiatr.

[16] Powlishta KK, Dras DD Von, Stanford A, Carr DB, Tsering C, Miller JP (2002). The clock drawing test is a poor screen for very mild dementia. Neurology.

[17] Spenciere B, Alves H, Charchat-Fichman H (2017). Scoring Systems for the Clock Drawing Test: a historical review. Dement Neuropsychol.

[18] Ryu S, Lee S, Song I, Kim Y, Lee K (2009). Qualitative Analyses of Clock Drawings in Mild Cognitive Impairment. Alzheimer Dement.

[19] Parsey C.M., Schmitter-Edgecombe M. (2011). Quantitative and Qualitative analyses of the Clock Drawing Test in Mild Cognitive Impairment and Alzheimer Disease: evaluation of a Modified Scoring System. J Geriatr Psychiatry Neurol.

[20] Lee AY, Kim JS, Choi BH, Sohn EH (2009). Characteristics of Clock Drawing Test (CDT) errors by the dementia type: quantitative and qualitative analyses. Arch Gerontol Geriatr.

[21] Suhr J, Grace J, Allen J, Nadler J, McKenna M (1998). Quantitative and qualitative performance of stroke versus normal elderly on six clock drawing systems. Arch Clin Neuropshychol.

[22] Pettersen RC, Doody R, Kurz A, Mohs RC, Morris JC, Rabins PV, Ritchie K (2001). Current concepts in mild cognitive impairment. Arch Neuropshychol.

[23] Fabricio AT, Aprahamian I, Yassuda MS (2014). Qualitative analysis of the Clock Drawing Test by educational level and cognitive profile. Arq Neuropsiquiatr.

[24] Brucki SMD, Nitrini R, Caramelli P, Bertolucci PHF, Okamoto IH (2003). Suggestions for utilization of the Mini-Mental State Examination in Brazil. Arq Neuropsiquiatr.

[25] Folstein MF, Folstein SE, McHugh PR (1975). "Mini-mental state": a practical method for grading the mental state of patients for clinician. J Psychiat Res.

[26] Blair M, Kertesz A, McMonagle P, Davidson W, Bodi N (2006). Quantitative and qualitative analyses of clock drawing in frontotemporal dementia and Alzheimer's disease. J Int Neuropsychol Soc.

[27] Kitabayashi Y, Ueda H, Narumoto J, Nakamura K, Kita H, Fukui K (2001). Qualitative analyses of clock drawings in Alzheimer's disease and vascular dementia. Psychiatry Clin Neurosci.

[28] Silva AM, Peçanha E, Charchat-Fichman H, Oliveira RM, Correa J (2016). Estratégias de cópia da Figura Complexa de Rey por Crianças. Rev Neuropsicol LatinoAmer.

